# *‘They say we are money minded’* exploring experiences of formal private for-profit health providers towards contribution to pro-poor access in post conflict Northern Uganda

**DOI:** 10.1080/16549716.2021.1890929

**Published:** 2021-05-13

**Authors:** Justine Namakula, Suzanne Fustukian, Barbara McPake, Freddie Ssengooba

**Affiliations:** a Department of Health Policy Planning and Management, Makerere University School of Public Health, Kampala, Uganda; b Department of Global Health and Development, Queen Margaret University, Edinburgh, UK; c Nossal Institute for Global Health, University of Melbourne, Melbourne, Australia

**Keywords:** For- profit providers, post-conflict, poverty, mechanisms, pro-poor access, Northern Uganda

## Abstract

**Background**: The perception within literature and populace is that the private for-profit sector is for the rich only, and this characteristic results in behaviours that hinder advancement of Universal health coverage (UHC) goals. The context of Northern Uganda presents an opportunity for understanding how the private sector continues to thrive in settings with high poverty levels and history of conflict.

**Objective**: The study aimed at understanding access mechanisms employed by the formal private for-profit providers (FPFPs) to enable pro-poor access to health services in post conflict Northern Uganda.

**Methods**: Data collection was conducted in Gulu municipality in 2015 using Organisational survey of 45 registered formal private for-profit providers (FPFPs),10 life histories, and 13 key informant interviews. Descriptive statistics were generated for the quantitative findings whereas qualitative findings were analysed thematically.

**Results**: FPFPs pragmatically employed various access mechanisms and these included fee exemptions and provision of free services, fee reductions, use of loan books, breaking down doses and partial payments. Most mechanisms were preceded by managers’ subjective identification of the poor, while operationalisation heavily depended on the managers’ availability and trust between the provider and the customer. For a few FPFPs, partnerships with Non-governmental organisations (NGOs) and government enabled provision of free, albeit mainly preventive services, including immunisation, consultations, screening for blood pressure and family planning. Challenges such as quality issues, information asymmetry and standardisation of charges arose during implementation of the mechanisms.

**Conclusion**: The identification of the poor by the FPFPs was subjective and unsystematic. FPFPs implemented various innovations to ensure pro-poor access to health services. However, they face a continuous dilemma of balancing the profit maximization and altruism objectives. Implementation of some pro-poor mechanisms raises concerns included those related to quality and standardisation of pricing.

## Background

The private sector is recognised as an important source of health care provision in many low-and-middle income countries [1–6]. However, the general perception in literature available and among the populace is that the private for-profit sector is for the rich [7–9] and that it can deter progress towards achieving Universal health coverage (UHC) [10]. The above perception is partly derived from the prevailing deficiencies in technical quality for services provided in the private sector [11]. Other critics have also highlighted over dependence on out of pocket expenditure(OPP) which results in catastrophic health expenditures among the poor [8,12–14] and widens inequities in access to health care [6,9].

Post-conflict Northern Uganda presents a paradox because there is growing presence of the for-profit sector despite (1) the effects of the protracted conflict that made the region lag behind the rest of the country and (2) high levels of poverty in the region [15,16]. Therefore, this article, had two specific objectives; first, to explore how the Formal private for-Profit Providers (FPFPs) identify the poor people in Gulu municipality; second, to identify access mechanisms employed by FPFPs to promote pro-poor access to health services. This study focused on Formal private for-profit providers, which are herein abbreviated as FPFPs. In this study, FPFPs are defined as facilities that are annually registered with the regulatory bodies and therefore appear on the list of registered private for-profit providers in Gulu district.

The article draws from a PHD study which focused on health care markets in post conflict settings, using experiences of formal for-profit health providers (FPFPs) in Gulu. The PHD was embedded in a program of research called ReBUILD Consortium, whose aim was to understand post conflict reconstruction of the health system in Northern Uganda. The article contributes to on-going debates about whether private sector, particularly for-profit, should or should not be engaged in achievement of Universal Health Coverage (UHC). UHC emphasises reduction of catastrophic expenditure, provision of quality services and expansion of service package [17] as well as living no one behind.

## Socio-economic and health indicators for Northern Uganda

Northern Uganda experienced a 26-year protracted armed conflict between the government of Uganda and rebels known as the Lord’s Resistance Army (LRA), resulting in destruction of property and displacement which hindered livelihoods as well as destruction of social safety nets [18–21]. The population of Northern Uganda/Northern region which comprises of Acholi and Lango sub- regions is quite high and this can be assessed using household size with Acholi sub-region being (5.5) compared to the neighbouring region of Lango(5.1) and national average of 4.7 [16]. The region reported some improvements to 61% arising from support of the functional adult literacy (FAL) programme implemented by the Ministry of Gender and Social Development (MoGLSD) and partners [22]. However, it is still lower than the national average of 73.5% [16]. Furthermore, the Northern region, has consistently been the poorest region in the country in the last decade, with poverty levels above the national average [23,24]. Despite the high poverty levels, the Uganda National Household Survey (UNHS 2016/17) indicates that a high number of people in Northern Uganda (Lango 55%, Acholi 35%) sought treatment from a private hospital or clinic, while 6.4% and 7.2% of the population in Lango and Acholi sub-regions, respectively, sought care from private pharmacies. Those who sought care from a public health centre were 31% and 53.4% for Lango and Acholi sub-regions, respectively. Fewer people in the two sub-regions (3.9% and 4.6% respectively), however, visited government hospitals [16].

## Methods

### Study site and sample

This study is based on data collected from March to May 2015 in Gulu Municipality, Northern Uganda. Data collection methods included a survey of 45 FPFPs, Organisational life histories (10) with selected categories of FPFPs in Gulu Municipality and key informant interviews (13) with Regulators at district level and directors of professional councils and bodies at national level, Health workers in other FPFPs, and representatives of other providers e.g. Public and PNFP.

For the organisational survey, a list of all the registered FPFPs, which was provided by the regulators who work with in the district health office in Gulu district, was used as a sampling frame. All the organisations that appeared on the sampling frame were visited for an interview. Out of the 60 organisations that appeared on the list, only 45 participated in the survey. The remaining 15 were excluded from the study for having declined to participate (12) and having moved business or having closed business despite being on the list (3).

The 10 FPFP units that participated in the organisational life history interviews were selected based on criteria derived from results of the preliminary organizational survey. In particular, the selection was based on two parameters; age of/length of stay in business and category of organisation. As a result, five old and five young FPFPs of varying categories were included for life histories. The ‘old’ and young organisations were those that had existed in business within the area for more than 10 years (>10) or less than 10 years, respectively. Facility managers of the selected sub-units of the FPFPs who were to participate in life-history interviews were selected based on the position they held in the FPFPs (either as managers or owners of businesses) Table 1.

**Table 1.** Social economic indicators for Northern Uganda

Year	Indicators	Region			
		Northern Uganda	Acholi	Lango sub-region	National average
2012/13	Household size	5	5	5	5
	Literacy	60	67.7	71.4	69.8
	Poverty estimates	43.7	Ranged from 40.1–55.0	–	19
2016/17	Average Household size	–	5.5	5.1	4.7
	Literacy levels (%)	–	59.7	77.6	73.5
	Poverty estimates (%)	33	34.7	17.6	21

Source [15,16,25,26].

Participants for key informant interviews were selected purposively based on their experiences derived from having worked in the region for a long time (above 10 years), working in other facilities in the market in Gulu or in positions of authority in relation to the supervision of FPFPs.

### Data collection

The organisational survey was conducted by an interviewer using a structured questionnaire. The purpose of conducting the survey was twofold – first, to establish the characteristics of the formal private for-profit organisations in Gulu municipality in the post-conflict period and, secondly, to facilitate ‘case screening’ [27] of FPFPs for organisational life history interviews. Case screening refers to the process of selecting participants using data provided by a survey but based on predetermined criteria. Managers or any other person delegated by the manager participated in the survey.

Key informant interviews were conducted using a semi-structured interview guide. During the life history interviews, a horizontal line (a timeline) was drawn on a paper and participants were asked to record key events in the life of their businesses. The timeline was used to probe for growth dimensions, challenges, coping strategies and decisions along the life of the business during and after conflict.

### Data analysis

Quantitative data were analysed using SPSS version 17 whereas qualitative data were coded using ATLAS ti version 7.0 and then thematically analysed.

## Results

The mini-organisational survey showed a variety of categories of FPFPs in Gulu municipality. These included clinics, clinics with laboratory sections, pharmacies (exclusively retail or wholesale, a mixture of wholesale and retail), stand-alone laboratories, medical centres, private hospitals, insurance company clinics, and others. Others included drug shops, imaging, and medical X-ray centres, ‘health centres’ IIs and NGO clinics. All health centres that were operating on a for-profit basis were at the HC II level. Ideally, the term ‘health centre II’ is common nomenclature under the public health system in Uganda. It refers to the first level of interaction between the formal health sector and communities. The health centre II is expected to offer a set of services. These include preventive, promotive, and curative services (mainly Out- Patient). Antenatal services may also be available [28]. Some of the FPFPs can acquire the same title if they satisfy the requirements of accreditation process for this level. As illustrated in Table 2, the majority of the FPFPs surveyed were clinics (18), followed by drug shops and medical centres (7). In addition, there was only one of each of the other categories of FPFPs surveyed. The majority of the FPFPs were located either along relatively busy streets or on the outskirts of Gulu municipality.

**Table 2.** Categories of FPFPs

Code	Organization	Frequency	Per cent	Cumulative Per cent
1	Clinic	18	40.0	40.0
2	Drug shop	8	17.8	93.4
3	Medical centre	7	15.6	55.6
4	Pharmacy	5	11.1	66.7
5	Laboratory	2	4.4	71.1
6	Private hospital	1	2.2	73.3
7	Insurance company clinic	1	2.2	75.6
8	X-ray and scan centre	1	2.2	95.6
9	NGO clinic	1	2.2	97.8
10	HC II (health centre level 2)	1	2.2	100
	Total	45	**100.0**	

## Identification of the poor people by the FPFPs

Before implementing the various mechanisms, the managers of FPFPs pragmatically applied a subjective criterion to identify the poor people. During analysis, the criteria was categorised into five main themes namely appearance of a person, perceived inability to pay bills, perceived level of politeness and place of residence and use of coins to pay for services. Consequently, individuals who appeared dirty on arrival at the facility, who said they were unable to pay bills, politely asked for a favour were categorised as being poor. Furthermore, those whose place of residence was located a long distance from Gulu town (rural Gulu) and those who brought in coins to pay a bill were perceived to be poor. The manager had to be present to make the decision on identification of the poor.

When you ask the patient to pay 2,000 shillings- [50 US cents] […] and they tell you they do not have money […] start pleading with you that I just walked to Gulu town from Ngai, or Opit village. […] if you do not help, they would probably have to walk back to Opit village without being treated or offered services. (P18: LH Manager Old FPFP)

[…] if patient came with coins rather than paper notes as payment for the service, it was a sign that the person has no money. (P20: LH Manager_ Young FPFP)

### Mechanisms employed by FPFPs to enable pro-poor access to health services

Various access mechanisms were employed by FPFPs to enable the poor to access health services in Gulu municipality. These included provision of free services and fee exemptions as well as and fee reductions. There were also some flexibility and practices around ensuring that payments were *‘manageable for clients namely* loans, fractions/partial doses and payment in instalments. The mechanisms are explained in detail in the next sections.

### Fee exemptions by FPFPs

**Figure 1.**
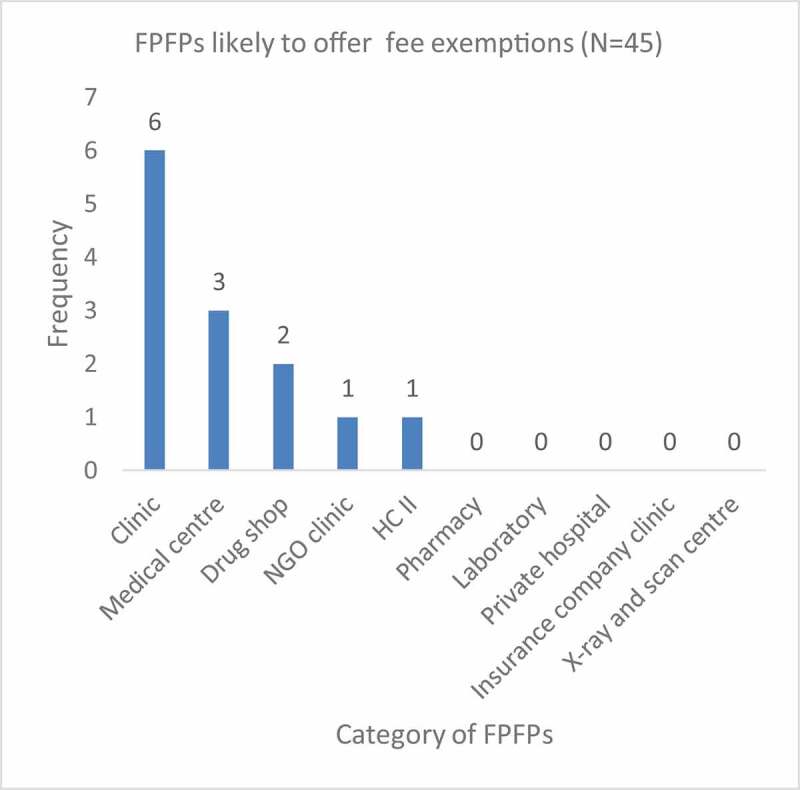
FPFPs likely to offer fee exemptions

All the 45 FPFPs interviewed except pharmacies, laboratories, private hospitals, and insurance company clinics, reported that they offered some fee exemptions (Figure 1). However, fee exemptions were most likely to be offered in clinics or medical centres compared to other categories of FPFPs. The categories exempted included the elderly, child mothers, adolescents, women within childbearing age (pregnant women) and people with disabilities (PWDs).

**Figure 2.**
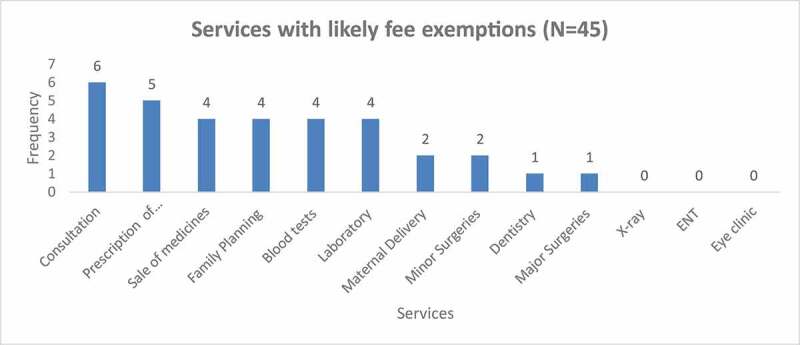
Services with likely fee exemptions

Survey results showed that fee exemptions were more likely to apply to basic support services such as consultation, prescription of medicines, sale of medicines, family planning and laboratory services However more complex services such as X-ray, ENT and eye clinic, major surgeries, minor surgeries, maternal deliveries, and dental services (Figure 2) were less likely to have fee exemptions. Further probing during qualitative interviews with FPFP managers revealed that there were other additional services to which fee exemptions applied namely, immunisation of children, family planning, HIV testing, screening for hepatitis B, diabetes screening and blood transfusion.

### Provision of free services and related motives

A few FPFPs, mainly the well-established and/or larger FPFPs reported provision of free immunisation services hence contributing to the broader coverage goals of the district. Free immunisation was enabled by government subsidies in the form of vaccines, fridges for storage and primary healthcare. In return, such FPFPs were expected to submit reports to the district offices. Other FPFPs reported provision of free services such as family planning, HIV testing, screening for hepatitis B, diabetes screening was enabled by partnerships with non-governmental agencies and education institutions within Gulu municipality. These services were made accessible during health camps whereas the partnerships were enabled due to lobbying undertaken by managers of certain facilities.

[…] we get the vaccines from the district and we do UNEPI [immunisation] here and then we give the district the report […]. (P15: LH_Manager Young FPFP)

In most cases FPFPs utilised the time when clients came in for free services to advertise for other paid services which were offered. For instance, when mothers came for free immunisation, they would be informed about other paediatric-related services while those who came up with positive tests during the health camps were advised to check with the facility for further treatment.

[…] If we find that the child also has malaria, we say that we can also give you anti- malarial[tablets] at a facility, and these are not for free. (P4: KI_HW other FPFP facility)

There was an interesting case of a manager who explained that their facility provided free services, for instance vaccinations, simply because they wanted to get rid of reagents that were about to expire.

### Price reduction and bargaining

Price reduction was another mechanism employed by FPFPs to enable pro-poor access to health services. FPFPs were more likely to offer price reductions for services such as sale of medicines, consultation and the prescription of medicines, blood tests and laboratory tests However, more complex services such as major surgeries, minor surgeries, ENT, laboratory and family planning X-ray, maternal deliveries did not attract any fee reduction.

**Figure 3.**
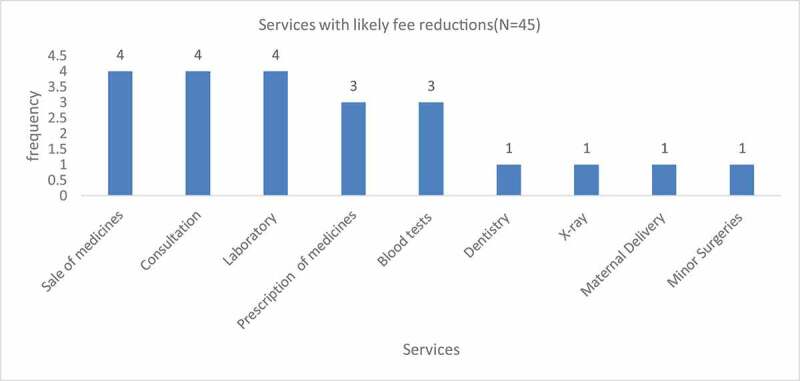
Services with likely fee reduction

In relation to the category of clients to whom fee reductions applied (Figure 3), FPFPs were most likely to offer fee reductions to the elderly (7), adolescents (5), Persons with disabilities (PWDs) (3), Persons Living with HIV/AIDS(PLWHAs) (3) and child mothers (3) and pregnant women. Additionally, other categories of clients, such as regular customers and uninsured patients, needy women and ‘those who cannot afford’ were also likely to receive price reductions. Only one FPFP reported a possibility of offering fee reductions to NGO staff whereas none of the FPFPs was likely to offer fee reductions to former abductees. When probed further, during the qualitative interviews, managers noted that majority of the NGO staff were insured hence rendering price reductions useless.

To protect themselves from making excessive losses when implementing fee reductions, FPFPs exercised price discrimination as well as bargaining. In relation to price discrimination some FPFPs reported varying fee charges across the perceived poor and perceived rich clients while ensuring that the latter made up for the shortfall. For instance, one of the managers reported reducing the price for those clients who were not under insurance cover while making the bill higher for those covered by insurance.

In some of the cases, price reductions emerged from a bargaining process between the manager and a client/patient to create a perception of a ‘win-win’ scenario for both the manager and the patient. However, in actual sense, clients still lost out and the facilities ended up making a profit. For instance, one manager reported haggling for the cost of an operation for a medical condition and noted that in the end, the patient went away with the satisfaction that they had negotiation a fair bargain, although the provider still got the expected price.

Overall, qualitative interviews also revealed that managers of the FPFP facilities were hesitant to publicize price reductions to minimize the chances of such incidents happening as well as misuse by prospective well-to do-clients.

‘They say we are money minded […] But we just do it quietly, otherwise even those with money will come and say that ‘we are being discriminated’. (P1: KI_ Manager Other FPFP Facility)

### Loan books and deferred payments

Loan books were commonly reported among ‘small’ providers as a strategy for maintaining clientele and ultimately dealing with competition with ‘big’ providers who have a greater market share. Loan books enabled the client to pay later and therefore were perceived to have reduced the burden/inconvenience on the client of paying the whole lumpsum fee. Whereas the presence of the manager was an enabler for decisions for loans or deferred payments to be made, the loan book was mainly an agreement between the manager and the client to whom the loan was given. Trust between the two parties therefore was crucial.

[…] we can give you your full dose, but you make a commitment to pay. For example, that woman you saw, we are giving her treatment for her child, and she has a loan book here. She can pay later […] (P4: KI Manager Other FPFP)

### Breaking down doses

Breaking down doses was a practice reported by the FPFPs to enable those who could not afford to pay for the full dose as a lumpsum. This was particularly common for malaria doses and was mainly reported by managers of smaller FPFPs. Under this arrangement, a client would be given a portion of the dose that they could afford on the assumption that they would come back the next day with money for the next dose. However, this raised quality issues if clients were unable to return.

[…] we tell them […] we have given you for two days because of the money you have. You can come back for the remaining part of the dose when you get the rest of the money […] there are some who go and don’t come back, and they will not heal because of the incomplete doses but I would not have lost money. (P2: KI Manager Other FPFP)

## Discussion

The article has shown that the implementation of any equity mechanism requires by FPFPs some measures of identification of the poor. Some of the parameters used by managers to identify the poor have a specific reference to conflict settings, for example, inability to pay bills is also indicated in the framework of vulnerability as part of the long-term effects of conflict which arise from destruction of livelihoods of populations [29]. Other scholars have termed this situation as ‘security-development nexus’ or the poverty- conflict nexus. This implies that there is a bi-directional relationship between poverty and conflict whereby poverty and conflict can reinforce or potentially lead to one another [30,31]. Another key finding was that application of the parameters for identification of the poor was not systematically applied across FPFPs and mainly depended on the presence and the mood of the manager. Hence, some of the extremely poor are likely to miss out on the opportunity to qualify for the free services or a reduction in price. There is, therefore, a need for a more systematic way of identifying the poor within the for-profit providers [32].

Our findings provide evidence about innovations devised by FPFPs to gain social acceptability and to contribute to the advancement of UHC goals. This can also be perceived as a contribution to the *know-how* knowledge type, which presents guidance on local operational knowledge about good practices by the formal for-profit private providers [33]. Furthermore, these findings are in contrast with the general impression within the literature and common perception amongst the populace that the private for-profit sector [only] exploits the population by making huge profit and deter achievement of UHC goals because it reaps off patients [8,12].

Notwithstanding, the findings about pro-poor mechanisms implemented by FPFPs further provide evidence about the FPFPs’ potential to act as social entrepreneurs by acting for a social cause [34]. This information contributes to debates around potential of the private sector to contribute towards public good [10]. Some of the mechanisms enabled a reduction in the financial burden that would result from the clients paying at one go while some flexibility and practices ensured that payments were manageable for clients. These included loans, fractions/partial doses, and payment in instalments. Some of these mechanisms are similar to those found in some previous studies conducted among informal providers in relatively stable contexts such as India and Bangladesh [35].

Some of the mechanisms for instance breaking down doses created dilemmas- where as they stretched the burden of out of pocket payment (OOP) over a period, hence making it more manageable for clients, implications for quality reflected through drug resistance due to failure of the clients return for completion of their dose. Related concerns about concern about quality of services in the for-profit sector in relation to breaking down doses has also been highlighted elsewhere [35]. Doherty highlights the importance of strong regulation of the for- profit private sector by governments in east and Southern Africa in relation to prohibition distortions in quality as well as price of health services among other disadvantages brought about by the for-profit sector [36].

The mechanisms of extending loans to clients was based on trust between the client and the owner of the FPFP, which, according to Bloom and colleagues [37] reflects ‘social-contractual relationship’ between the providers and the clients. Trust is important for the effective performance of health systems, given that it creates a feeling of satisfaction in connection with both clients’ needs/expectations and providers’ expectations [37,38]. Another study conducted earlier on the private sector in the conflict-affected region of Northern Africa also found trust to be an important aspect of business [39].

Unlike the young FPFPs, the older FPFPs were more likely to provide free services through partnerships with government agencies and with NGOs. These partnerships with governments although limited to a few FPFPs, not only enabled the FPFPs to contribute towards the broader coverage goal for UHC but also provided an opportunity for advertisement of other services to increase their perceived market share. This finding provides evidence for possible partnerships with private for profit sector in post conflict Northern Uganda, a bold step from breaking the 40 decade of including only the Not-for Profit sector [28,40].

The findings have shown that FPFPs would like to maintain a good image among the public by ensuring that they enable access to healthcare services by the poor. However, they continuously face a dilemma of balancing the optimization of their incomes with the altruism objectives and ultimately moving along the extreme continuum of social entrepreneurship. This dilemma was reflected in; the hesitance FPFPs to publicise some mechanisms; FPFP whereas their motivation to implement as well as actual operationalisation of other mechanisms. For instance, some FPFPs leveraged the opportunity made available by the provision of free services, which was enabled by partnerships with other sectors, to market the other services that they provided in-house. Furthermore, findings indicated that the package of services selected for fee exemptions was narrow, and mainly focused on prevention and less on some most critical services, such as diagnostic and maternal deliveries. Therefore, our findings suggest that if FPFPs are to be leveraged in terms of UHC, there is need for a more comprehensive and clearer package of service.

Notably, the findings indicated that negotiation, a key process for one of the mechanisms- that is- price reduction has limitations, since some managers reported exaggerating the price of treatment while withholding information from clients, which is evidence of false billing, one of the unethical business practices exercised by private sector in sub-Saharan Africa, mainly driven by the ‘pursuit of excess profits’. Other unethical business practices by for-profit sector include over-servicing, collusion, false billing, price gouging, and unlicensed practice [41]. Furthermore, the practice of withholding information exercised by some of the managers rhymes with the New Institution economics concept of information asymmetry [42].Information asymmetry is a situation where one party in a transaction has more information than the other and uses this position to take advantage of the situation [42,43].

### Limitations of the study

The findings of this study need to be considered bearing in mind some limitations. The study had a supply-side bias hence does not include clients’ views and how they perceived/identified a poor person. The study also potentially suffered from a social acceptability/desirability because it relied on provider self reports of their characteristics and behaviour particularly in relation to actions for enabling access to the poor [44]. Therefore, it is impossible to cannot rule-out the possibility of providers either over-reporting good behaviour or under reporting bad behaviour [45]. Lastly, there is limited generalizability of findings due to the relatively small number of organizations that were involved in the organisational survey and qualitative interviews.

## Conclusions

This study has highlighted experiences of FPFPs in relation to enabling the poor to access health services. The identification of the poor by the FPFPs was subjective and often unsystematic. FPFPs implemented various innovations to ensure pro-poor access to health services. However, they face a continuous dilemma of balancing the profit maximization and altruism objectives. Implementation of some pro-poor mechanisms raises concerns included those related to quality and standardisation of pricing.

## Recommendations

Based on the above findings, the following recommendations were derived. Regarding identification of the poor, there is need for a more systematic way of identifying the poor within the for-profit providers to ensure that some of the poor do not miss-out. Increased regulation can mitigate the quality and charging related challenges arising from some mechanisms. The package of free services needs to be broadened to include curative services. Support to FPFPs should be expanded to cover more providers and sustained to enable their contribution to wider Universal Coverage goals

## Data Availability

Data and materials are available as supplementary information on request.

## Paper context

The populace and pro-Universal Health coverage supporters expect the private for-profit sector to serve the rich only. This paper provides evidence about private providers’ access mechanisms to ensure pro-poor relevance and contribute to a social mission. However, balancing the profit maximization and altruism objectives is a continuous dilemma. The sub-sector’s contribution can be strengthened through increased support, partnership by government, regulation and possible investments by donors.
